# China’s fertility change: an analysis with multiple measures

**DOI:** 10.1186/s12963-022-00290-7

**Published:** 2022-03-31

**Authors:** Shucai Yang, Quanbao Jiang, Jesús J. Sánchez-Barricarte

**Affiliations:** 1 School of Public Policy and Administration, Xi’an Jiaotong University, Xi’an, China; 2 Institute for Population and Development Studies, Xi’an Jiaotong University, Xi’an, China; 3 Carlos III University of Madrid, Getafe, Spain

**Keywords:** Total fertility rate, Tempo effect, Parity progression ratio, Completed cohort fertility rate, Indirect estimation

## Abstract

**Background:**

The period fertility in China has declined to very low levels, and the completed cohort fertility rate (CFR) has also decreased significantly. However, the exact fertility rate remains controversial. While the tempo effect has played a significant role in China’s period fertility decline, child underreporting has to be taken into consideration in China’s fertility research.

**Methods:**

By using the census data from 1982 to 2010, and the 1% population sample survey data from 1995 to 2015, we systematically analyzed China’s fertility and its trends since the 1980s using period fertility measures, adjusted period fertility measures, cohort fertility measures, and indirect estimation methods.

**Results:**

The results show that marriage postponement significantly affects the TFR decline. Even after eliminating the tempo and parity structure effect, the adjusted TFR has fallen below 1.5, and the first-order fertility rate dropped to 0.9 in 2015. The CFR for women aged 45–49 declined from 5.37 in 1982 to 1.62 in 2015 mainly because of a decrease in fourth and higher-order births from 1982 to 1990, a decrease in second and third births from 1990 to 2000, and a decrease in second births from 2000 to 2015. Indirect estimation methods yielded a TFR in the range of 1.5–1.6 for the period 2000–2010 and an average TFR of 1.49 for the period 2011–2020.

**Conclusions:**

The traditional norm of universal marriage and childbearing for Chinese women is changing. China’s fertility has been steadily declining, as measured by both period and cohort indicators. Following the historical change, fertility may continue to decline even after introducing the universal three-child policy in China in 2021.

## Introduction

China’s fertility level has continuously declined over the past decades. The total fertility rate (TFR) decreased from 5.81 in 1970 to 2.75 in 1979. In the 1980s, the TFR fluctuated above the replacement level. Since the 1990s, the fertility rate has declined to below the replacement level [1]. The 2010 and 2020 censuses yielded TFRs of 1.18 and 1.30, respectively [2, 3]. China’s National Bureau of Statistics (NBS) reported the annual births of 10.62 million for 2021 [4], a sharp decline of 11.50 percent compared to the 12 million births enumerated in China’s 2020 census. While China’s registered birth rates are widely considered to be underestimated owing to child underreporting [5, 6], it is generally recognized that China’s fertility has decreased to very low levels since the 1990s [1, 7–9]. As with other countries that have experienced population transitions, China’s fertility decreased to the replacement level and continued to decrease to extremely low levels, rather than being sustained at the replacement level as expected. Following the TFR of 1.3 in 2020 census, the decline in births in 2021 indicated that the fertility level decreased further.

In China’s fertility decline over the past decades, marriage and childbearing postponement played a considerable role. Research in western countries shows that marriage postponement is a major driving factor of TFR decline [10–14]. Because of the Marriage Law enacted in 1981, the age at first marriage in the early 1980s was lower than in the 1970s, and it increased steadily after 1986 [15]. The average age at first marriage has been increasing since the 1990s, despite some fluctuations [16]. The rising age at first marriage resulted in the declining age-specific proportion of currently married women among all women in this age group. Whether using census data [1] or adjusted data [5], the decrease in the proportion of currently married women can account for 40% of the fertility decline between 1990 and 2000. The TFRs for 2010 and 2015, standardized with marriage proportions in 1990, are 0.42 and 0.53 higher than the observations [16].

Some studies have reported the effect of tempo and parity change on fertility. The parity progression ratio—a measure proposed by Feeney (1985) [17], Ma et al. (1986) [18], and Feeney and Yu (1987) [19]—not only accounts for the effect of parity structure but is also less distorted by the tempo effect. Believing that period TFR can be distorted by the tempo effect resulting from childbearing age variation, Bongaarts and Feeney (1998) [20] proposed a counterfactual measure, the adjusted total fertility rate, which is equal to the TFR that would have been observed in a calendar year if there had been no delay of childbearing. According to studies on China, the tempo effect of late marriage and late childbearing caused TFR to decline to 0.2–0.4 in the 1970s, but had no significant effect on the TFR in the 1980s. It also caused decline of the TFR to be in the range of 0.1–0.2 in the 1990s [21]. Tempo effect adjustment resulted in higher estimates of China’s TFR [21–23].

Chinese women’s completed cohort fertility rate (CFR) also exhibits a significantly decreasing trend. Census data show that the average number of children born to women aged 45–49 years decreased from 5.37 in 1982 to 1.84 in 2010 [2, 24]. The cohort of women born in 1976–1986 will have a CFR of 1.7 [7]. During the transition from a higher level of fertility to the replacement level, the decline in third and higher-order births was the dominant factor; in the phase of decline of fertility below the replacement level, the decline in first- and second-order births was the main factor [25]. Therefore, it is important to examine the decline in cohort fertility of the Chinese population since the 1980s according to parity structure.

Child underreporting is an issue that has to be considered in China’s fertility research [6]. Studies show that births in China in the 1990s and 2000 were underreported [26, 27]. However, more researchers have recognized possible overreporting in annual birth data published by the National Bureau of Statistics of China in recent years [6, 7, 28, 29]. Studies also show inadequate evidence to support the presence of large-scale, continuous underreporting in China [30, 31]. There are many techniques for the indirect estimation of fertility, including variable-r, P/F ratio, reverse survival analysis, and prediction modeling. Different methods may yield different results [29, 32–35].

The decline in fertility and births in China has been a concern for the government. After a steep decline of annual births from 14.65 million from 2019 to 12.00 million 2020, the number of annual births further declined 10.62 million, which has stirred up hot debate in China. The pandemic of COVID-19 accounted for partly the decline in birth numbers, but the main driver was the fertility decline due to low fertility intention and postponement in childbearing [36]. To counteract a further decline in fertility, the Chinese government introduced the universal two-child policy in 2016, which allowed all married couples to have two children [37]. However, this policy has not triggered the expected baby boom; the number of births in 2017, 2018, and 2019 was 17.23, 15.23, and 14.65 million, respectively. The decreasing fertility rates and newborn populations attracted extensive discussion in the scholarly community [1, 9, 38, 39]. China’s 2020 population census enumerated a total of 12.00 million births and a TFR of 1.3. Following this census, the Chinese government introduced the universal three-child policy and further required to optimize the fertility policy to promote long-term balanced development of the population, which include eliminating restrictive measures like social maintenance fees (fines for out-of-quota births), removing related penalties, and implementing pronatalist policies [40]. However, in a context of low fertility desire, these polices and requirements received little response from the people and would fail to increase the fertility level.

In the wake of the universal three-child policy and pessimistic fertility prospect, we expect to systemically depict the fertility trend over the past four decades and provide some reference for future study. We investigate the effect of marriage and childbearing postponement on China’s fertility decline, depict the fertility change trend after eliminating the tempo effects and examine the historical course of China’s cohort fertility decline. We apply period fertility measures, adjusted period fertility measures, cohort fertility measures, and indirect estimation methods to data from censuses, and 1% population sample surveys and annual one in thousand population sample survey since 1982.

## Methods

This study analyzes fertility using the following four groups of measures and methods: (1) period fertility measures, including period total fertility rate (TFR), age-specific fertility rate (ASFR), mean age at childbearing (MAC), total marital fertility rate (TMFR), ratio of TFR to TMFR, standardized TFR, and decomposition of TFR variations; (2) adjusted period fertility measures, including period parity progression ratios (PPPRs) and period total parity progression fertility rates (PTPPFR), tempo-adjusted TFR, and tempo- and parity-adjusted TFR; (3) cohort fertility measures, including the proportion of women with at least N births, completed cohort fertility rate (CFR), cohort parity progression ratio, and decomposition of CFR changes; (4) indirect estimation methods, including variable-r method, P/F ratio method, reverse survival method, and projection simulation method. We didn’t list all the detailed formulas here due to space limit. Please refer to “Appendix 1” for the details of these methods.

## Data

In China, several government agencies provide demographic statistics. The National Health Commission (into which the former Population and Family Planning Commission was merged) is responsible for implementing and monitoring family planning policies and provides statistics of annual births. As births and birth rates are measures for assessing the performance of family planning work, the birth statistics provided by the family planning agency may be unreliable [26, 41]. China’s Public Security Bureau is responsible for household registration. Although household registration regulations require newborns to be registered within one month, delays in registration of births often occur [42, 43]. The 2010 census shows that 13 million people were without household registration in the public security system. Most of them were out-of-quota births under the family planning system [44].

China’s fertility decline was accompanied by data quality issues, particularly large-scale underreporting of births [6, 45]. Studies around 2000 show significant underreporting in the 1990 and 2000 censuses [26, 27]. The large-scale rural–urban migration and the stringent family planning policies resulted in severe underreporting of births [28, 46, 47]. However, the underreporting of births has not been adequately addressed in China’s fertility research. Reports show that 19% of the population aged 0–4 years was not reported in the 2000 census [6]. The registered TFR in the 2000 census was 1.22, but the fertility rate used for internal purposes by China’s National Bureau of Statistics was 1.40 [7]. Since 2000, however, more researchers have recognized the possibility of overreporting in China’s birth data [6, 7, 28, 29]. Researchers are also arguing for inadequate evidence to support the existence of large-scale, continuous underreporting in China [30, 31]. Judging from the 2010 census, a common issue with the demographic estimation and prediction from 1990–2010 is the overestimation of births and fertility. Statistical adjustments were made to adjust for overestimation, but the error resulting from such adjustment was greater than the error of the original census data, resulting in a seriously misleading overview of fertility trends [31]. In the absence of other reliable data, China’s census data are the most reliable source of demographic data. This is why we still use the census data to evaluate China’s fertility rate. With the second demographic transition, China also faces delays in marriage and childbirth, so the TFR is lower than women’s actual fertility over their lifetime, adding to people’s distrust of fertility data. For this reason, we used both period and cohort perspectives and used multiple indicators to re-estimate China’s historical fertility level so that we can approximately understand China’s fertility level.

We used the tabulated data of 1982, 1990, 2000, and 2010 censuses and the 1% national population sample surveys in 1995, 2005, and 2015. The 0.95‰ case data contained in the 2000 census were also used. The mean ages at childbearing in 1986, 1996, and 2011 were computed using the age-specific fertility data tabulated in the *China Population and Employment Statistical Yearbook 2018*. This study did not adjust the tabulated data. The 2020 population census has been completed. However, except for publicly announced birth number and TFR, we could not get further information from 2020 population census and so we didn’t include in-depth analysis on 2020 data in this paper.

## Results

### Period fertility measures

#### Total fertility rate

**Fig. 1:**
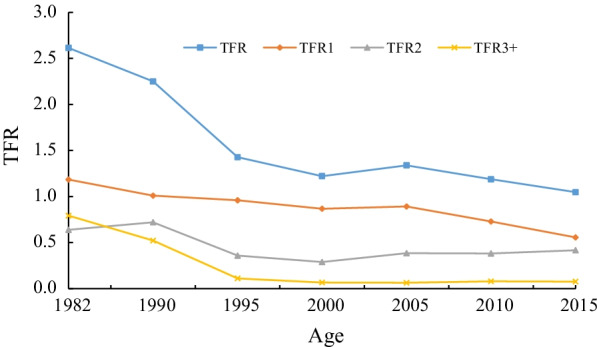
TFRs by birth order in China, 1982–2015

Figure 1 presents the TFR data. The TFR has been declining since the 1980s and has declined to below the replacement level since the 1990s. The first-order TFR has kept declining. The second-order TFR initially declined, then began to d increase after 2000. The third- and higher-order TFR declined but remained stable after 2000. During 1982–1995, the decline in the TFR of the third- and higher-order births was the largest contributor to the TFR decline in China. Between 1995 and 2015, the decline in first-order TFR was the largest contributor to the decline in TFR.

We calculated the singulate mean age at marriage (SMAM), as shown in Table 1. The SMAM showed a downward trend from 1982 to 1990, and an upward trend from 1995 to 2015. Prior to 1980s, the “later, longer, fewer” family planning policy required that the minimum age for women to marry was 23 years old. The SMAM for women in 1982 was 22.38. The minimum legal marriage age for women in the 1981 version of the Marriage Law was 20 years, lower than that required by the “later, longer, fewer” policy. This decline in age at first marriage required contributed to the fertility rebound in the early 1980s. The SMAM for women had a downward trend in the several years after 1982. With the development of the social economy, there gradually showed a trend of late marriage in the 1990s. This is why the SMAM declined first and then increased, as shown in Table 1.

**Table 1:** Age-specific fertility rate, MAC, and SMAM by birth order

Year	ASFR (‰)							MAC	MAC1	MAC2	MAC3 +	SMAM
	15–19	20–24	25–29	30–34	35–39	40–44	45–49					
1982	6.12	144.73	235.74	85.70	32.90	14.31	3.26	27.98	25.26	27.25	32.64	22.38
1990	21.99	198.81	155.55	55.74	19.56	5.67	1.63	26.12	23.43	26.59	30.66	22.07
1995	10.89	154.07	91.84	26.50	5.71	1.58	0.63	25.22	23.81	27.40	30.43	22.58
2000	5.96	114.49	86.19	28.62	6.22	1.46	0.68	25.87	24.50	28.80	31.08	23.31
2005	6.34	114.46	91.70	40.22	10.98	2.05	0.77	26.41	24.59	29.79	31.58	23.55
2010	5.93	69.47	84.08	45.84	18.71	7.51	4.68	28.44	26.65	30.83	33.44	24.67
2015	9.19	54.96	74.31	45.31	18.60	5.37	3.11	28.48	26.63	30.21	32.56	25.39

Data source: The results for 1982–2015 were computed using tabulated age-specific fertility data from the censuses and 1% population sample surveys during the same period

#### Age-specific fertility rate

Table 1 presents the age-specific fertility rates. Overall, the fertility of the two age groups of women in their prime childbearing ages (20–24 and 25–29 years) kept declining. In 1982, women aged 25–29 years had the highest fertility rate. During the period 1990–2005, women aged 20–24 years had the highest fertility rate. After 2010, women aged 25–29 years regained the position of highest fertility rate. The fertility of women aged 30–49 declined after the 1980s, indicating the declining fertility rate of higher-order births. The fertility of this age group rebounded in 1995 and after 2000, indicating delayed marriage and increasing numbers of second births in China.

#### Mean age at childbearing

The mean age at childbearing (MAC) is presented in Table 1. The MAC initially decreased and then increased. In 1982, the MAC was 27.98 years then dropped to 25.22 in 1995 before rising to 28.48 in 2015. Despite a decline from 1982 to 1990, the MAC for first and second births has steadily increased since 1990. This indicates the effect of delayed marriage. Between 1982 and 1995, the MAC for third- and higher-order births decreased. This is due to the interaction of two factors: (1) the declined fertility and proportion of the fourth-, fifth-, and higher-order births and (2) delayed childbearing. The effect of delayed childbearing can be seen from the fact that, since 1995, when the TFR of third births stabilized, the mean ages at third- and higher-order childbirths have gradually increased.

Variations in MAC are caused by the interaction of parity structure and parity-specific childbearing age. Between 1982 and 1995, the TFR of third- and higher-order births decreased rapidly, as did the proportion of third- and higher-order births to total births, resulting in declining mean ages at childbearing. After that, the effect of delayed childbearing started to become apparent, resulting in rising mean ages at childbearing. This pattern of childbearing age variations has been extensively verified in Western countries [48].

#### Total marital fertility rate

The TMFRs presented in Table 2 have been declining with time. The TFR to TMFR ratio measures the average age-specific proportions ever married weighted by the age-specific marital fertility rates. The average proportion ever married was 0.75 in 1990, declined to 0.54 in 2000 and declined further to 0.49 in 2015. Although universal marriage is still a norm for Chinese women, young adults have been delaying marriage since the 1990s. As childbearing within marriage is still the social norm for Chinese people, delayed marriage results in delayed childbearing and declined fertility.

**Table 2:** TMFRs and TFR/ TMFR

Year	TMFR	TMFR_1_	TMFR_2_	TMFR_3+_	TFR/TMFR
1990	2.99	1.53	0.90	0.56	0.75
1995	2.36	1.77	0.46	0.12	0.61
2000	2.25	1.80	0.37	0.08	0.54
2005	2.38	1.83	0.48	0.07	0.56
2010	2.13	1.51	0.52	0.10	0.56
2015	2.12	1.35	0.67	0.10	0.49

Data source: The results for 1990–2015 were computed using tabulated age-specific fertility data from the censuses and 1% population sample surveys during the same period

The computed results of TMFR for women aged 15–19 were overestimated owing to underreporting of their marriage in the censuses. Therefore, data for women aged 20–49 are included in TMFR. To facilitate comparison, the TFRs in the TFR/TMFR are also for women aged 20–49 years

#### Standardized TFR

Table 3 shows the standardized TFRs using different age-specific proportions ever married. The first column in the table shows the years in which the proportions ever married were used to standardize the fertility data. As shown in Table 3, when the fertility data are standardized with the age-specific proportions ever married in a given year, the standardized TFRs before that year are lower than the observed TFRs, and the standardized TFRs after that year are higher than the observed TFRs. The standardized TFRs using different age-specific proportions ever married show a decreasing trend, as indicated in the table by column. The data reflect the effect of increasing proportions of unmarried women, delayed marriage, and delayed childbearing on the decreasing TFRs.

**Table 3:** Standardized TFRs

Year	1982	1990	2000	2010	2015
Surveyed	2.61	2.25	1.22	1.18	1.05
1982	2.61	2.17	1.47	1.56	1.55
1990	2.68	2.25	1.54	1.61	1.60
2000	2.37	1.86	1.22	1.36	1.33
2010	2.05	1.59	1.03	1.18	1.16
2015	1.88	1.43	0.92	1.08	1.05

Data source: as in Table 1

#### Decomposition of TFR changes

As shown by Jiang et al. (2019) [1], the TFR change can be decomposed into two factors: the change in marital fertility rate and the change in proportion ever married. Between 1990 and 2000, the TFR decreased by 1.03; the decline in marital fertility and marriage proportions contributed 59.20 percent and 40.80 percent, respectively, to the decline. The contributions of these two factors are almost 60% and 40%, respectively, whether using unadjusted census data [1] or adjusted census data [5]. During 2000–2010, the TFR decreased by 0.03. The decrease in marriage proportion reduced the TFR by 0.17, while the increase in marital fertility rate raised the TFR by 0.14. When marriage is delayed, the proportion of people who marry falls even lower, resulting in a lower TFR.

### Adjusted period fertility measures

#### Period parity progression fertility rates (PPPFR)

**Fig. 2:**
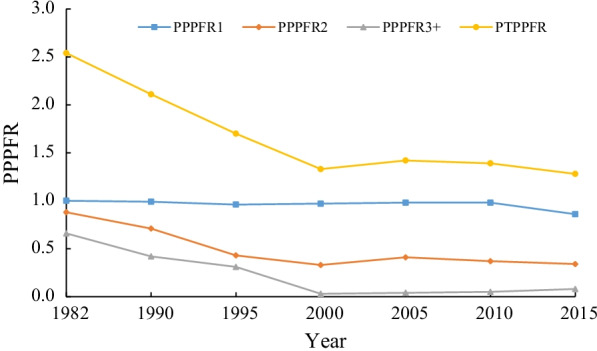
Period parity progression ratios by birth order in China, 1982–2015

Figure 2 shows the period parity progression fertility rate by birth order and period total parity progression fertility rates (PTPPFR). From 1982 to 2000, the PTPPFR decreased from 2.54 to 1.33. More specifically, the period parity progression fertility rate for second births decreased from 0.88 to 0.33 and from 0.44 to 0.03 for third births; the period total parity progression fertility rate for first births is nearly 1, indicating an extremely small proportion of childless women [49]. From 2000 to 2010, The PTPPFR exhibited no remarkable variation [50]. From 2010 to 2015, the period total parity progression fertility rate for first births decreased to 0.86, indicating a decreased first-order fertility rate and an increased proportion of childless women; the period total parity progression fertility rate of second births fell in the range of 0.3–0.4, roughly reflecting the latest level of second births.

#### Tempo- and parity-adjusted TFR

Table 4 shows the tempo-adjusted total fertility rates ($TFR^{*}$), parity-adjusted total fertility rates ($TFRp$), and tempo- and parity-adjusted total fertility rates ($TFRp^{*}$). The $TFR^{*}$ was 2.10, 1.48, and 1.11 in 1990, 2000, and 2015, respectively. The $TFRp$ was 2.26, 1.36, and 1.40 in 1990, 2000, and 2015, respectively. The sharp decline $TFRp$ during 1990–2000 is mainly because of the sharp decline in the $TFRp$ for second-, third- and higher-order births. The $TFRp$ increased slightly during 2000–2015. The $TFRp$ for first births decreased, while the $TFRp$ for second births increased. The $TFRp^{*}$ was 2.22, 1.49, and 1.42 in 1990, 2000, and 2015, respectively.

**Table 4:** Tempo- and parity-adjusted TFRs

Year	*TFR*	*TFR*_1_	*TFR*_2_	*TFR*_3+_	$TFR^{*}$	$TFR_{1}^{*}$	$TFR_{2}^{*}$	$TFR_{3 + }^{*}$
1990	2.25	1.01	0.72	0.52	2.10	0.94	0.62	0.55
2000	1.22	0.87	0.29	0.06	1.48	1.01	0.40	0.07
2015	1.05	0.56	0.42	0.08	1.11	0.63	0.40	0.08

Year	$TFRp$	$TFRp_{1}$	$TFRp_{2}$	$TFRp_{3 + }$	$TFRp^{*}$	$TFRp_{1}^{*}$	$TFRp_{2}^{*}$	$TFRp_{3 + }^{*}$
1990	2.26	0.99	0.74	0.54	2.22	0.98	0.68	0.55
2000	1.36	0.96	0.34	0.06	1.49	0.98	0.44	0.07
2015	1.40	0.87	0.45	0.08	1.42	0.90	0.44	0.08

Data source: The 1990 results were computed using the tabulated data of the 1990 census; the 2000 results were computed using the 0.95‰ case data contained in the 2000 census; the 2015 results were computed using the 1% population sample survey in 2015

Note that, the period parity progression fertility rate for first births was 0.86 in 2015 (Table 4), the $TFRp$ for first birth was 0.87, and the $TFRp^{*}$ for first birth was 0.9. Universal marriage and childbearing are the traditional norms for Chinese women, but the increasing proportion of women choosing to remain unmarried and childless is a trend deserving attention.

### Cohort fertility measures

#### The proportion of women aged 45–49 with at least N live births

Table 5 shows the proportion of women aged 45–49 with at least N live births since the 1980s. In 1982, the proportions of women aged 45–49 with at least four and five live births were 81% and 65%, respectively. In 1990, the proportions for women aged 45–49 dropped slightly compared to those in 1982. For women aged 45–49 in 2000, 78% had second births, and 38% of women had third births. The “one-and-a-half” or “two-child” policy was implemented in most regions of China [51], but there were about 200 million out-of-quota births under the family planning policy [52, 53]. When China began implementing the strict family planning policy in the 1980s, fertility declined rapidly, but second births remained a common choice.

**Table 5:** Proportion with at least N live births, Live births for different cohorts by year

Year	Proportion with at least N live births (%)					Live births for women aged		
	One	Two	Three	Four	Five	35–39	40–44	45–49
1982	0.98	0.96	0.91	0.81	0.65	3.80	4.64	5.37
1990	0.99	0.96	0.84	0.61	0.34	2.48	3.21	4.00
1995						2.08	2.44	3.05
2000	0.99	0.78	0.38	0.13	0.04	1.85	2.05	2.36
2005						1.67	1.91	2.09
2010						1.52	1.69	1.84
2015	0.96	0.52	0.11	0.02	0.01	1.46	1.54	1.62

Data source: as in Table 2. The data of the proportion with at least N live births in 1995, 2005 and 2010 are missing

#### Cohort parity progression ratio and CFR

Table 6 presents the cohort parity progression ratios for women aged 45–49. Between 1982 and 1990, the cohort parity progression ratios from third to fourth births and from fourth to higher-order births declined markedly. From 1990 to 2000, the cohort parity progression ratio to higher-order births declined. Furthermore, during 2000–2015, the progression ratios from first to second births and from second to third births declined significantly. In 2015, the progression ratio from first to second births was only 0.55.

**Table 6:** Parity progression ratios and the decomposition of the CFR changes

Year	PPR_0,1_	PPR_1,2_	PPR_2,3_	PPR_3,4_	CFR
1982	0.98	0.98	0.95	0.89	5.37
1990	0.99	0.97	0.87	0.72	4.00
2000	0.99	0.79	0.49	0.35	2.36
2015	0.96	0.55	0.21	0.20	1.62

	ΔCFR	dPPR_0,1_	dPPR_1,2_	dPPR_2,3_	dPPR_3,4+_
1982–1990	− 1.37	0.03	− 0.02	− 0.28	− 1.10
1990–2000	− 1.64	− 0.01	− 0.55	− 0.73	− 0.35
2000–2015	− 0.74	− 0.07	− 0.41	− 0.23	− 0.03

Data source: The 1982 results were computed using case data downloaded from the Integrated Public Use Microdata Series (IPUMS) of University of Minnesota; the 1990 results were computed using the live birth data contained in the 1990 census; the 2000 results were computed using the 0.95‰ case data contained in the 2000 census; the 2015 results were obtained using the 1% population sample survey in 2015

The decline in cohort parity progression ratios led to the decline in CFR. The cohort of women aged 45–49 had 5.37 live births in 1982. This figure decreased to 2.36 in 2000, 1.84 in 2010, and 1.62 in 2015.

#### Decomposition of CFR changes

Table 6 presents the decomposition of the changes in the CFR during 1982–1990, 1990–2000, and 2000–2015. The CFR declined by 1.37, 1.64, and 0.74 in these three periods. During 1982–1990, the CFR declined by 1.10 because of the decreased progression ratios from third to fourth and higher-order births. During 1990–2000, the primary driver of the CFR decline is the decline in the progression ratio from second to third births, contributing 0.73 to the decline. During 2000–2015, the primary driver of the CFR decline was the changed progression ratio from first to second births, contributing 0.41 to the decline. During the transition from higher fertility levels to the replacement level, the decline in the fertility rate of third and higher-order births was the leading factor; in the stage of fertility decline to below replacement levels, the decline in the fertility rate of first and second births was the primary factor [25].

### Fertility levels by indirect estimation methods

Table 7 presents TFRs obtained using the variable-r method, P/F ratio method, and reverse survival method.

**Table 7:** Fertility estimates using different methods

Year	Variable-r	Variable-r_2	P/F (a census)	P/F(two censuses)	Reverse survival
1982					2.95
1990			2.29		2.63
2000			1.43		1.54
2010			1.50		
1990–2000	1.58	1.74		1.35	
2000–2010	1.68	1.52		1.32	

The first variable-r method in the second column uses the census data directly; the second variable-r method in the third column uses the census data by increasing the population aged 0–9 by 10 percent in 2000

Concerning the results of the variable-r method, the underreporting of children in the 2000 census was more serious relative to that in 1990 and may affect the results. In this article, we applied the variable-r method directly with the census data, and the adjusted data that increased the population aged 0–9 in 2000 by 10%. The TFR estimated by variable-r method for 1990–2000 using the census data was 1.58 and was 1.74 using the adjusted data. The TFR for 2000–2010 was 1.68 when using census data directly and 1.52 when using the adjusted data. The results are similar to those by Cai (2008) [29], Chen (2015) [33], and Zhao (2015) [35].

The TFR estimates by the P/F ratio method with data from a single census are 2.29, 1.43, and 1.50, for 1990, 2000, and 2010, respectively. The average TFRs for 1990–2000 and 2000–2010 estimated by applying the P/F ratio method to data from two censuses [54] are 1.35 and 1.32, respectively.

The TFR estimates for 1982, 1990, and 2000 using the reverse survival method are higher than the figures obtained in the censuses. The TFR estimate for 2000 is 1.54, much higher than the 1.22 obtained by the census data.

Table 8 presents the projection simulation results. The “TFR in Yearbook” in the second column are obtained by summing over the age-specific fertility rates from the annual population change sample surveys included in China Annual Statistical Yearbook. Since early 1990s, the Chinese government no longer provided the TFR data, but the age-specific fertility rates from annual population sample survey were listed in annual yearbooks. The TFRs yielded by annual sample surveys since 1995 are generally below 1.5. With the age-specific fertility rates, and population census data, we projected annual births and obtained the “Birth estimate” data in the third column. Generally, the birth estimate was lower than that officially announced as “Published births” in the fourth column. We multiplied the “TFR in Yearbook” by the scaling ratio of “Published births” to “Birth estimate” and obtained the “Estimated TFR” in the fifth column. Except for 2017, the estimated TFR falls in the range of 1.5–1.6 for the years 2000–2010 and is generally below 1.5 for the years 2010–2020. Although the estimated TFR for 2016 and 2017 are higher than 1.6 due to the baby boom brought about by the universal two-child policy. However, the TFR fell below 1.5 after 2018, to 1.3 in the 2020, and further to 1.07 in 2021. The ultra-low fertility in the past several years was partly a result of the pandemic of COVID-19, but was also a reflection of China’s low fertility level.

**Table 8:** Birth and fertility estimates by projection simulation method

Year	TFR in yearbook	Birth estimate (million)	Published births (million)	Estimated TFR
1991	1.97	22.08	22.65	2.02
1992	1.83	20.97	21.25	1.86
1993	1.69	19.68	21.32	1.84
1994	1.56	18.35	21.10	1.79
1995	1.43	16.75	20.63	1.76
1996	1.44	16.79	20.67	1.78
1997	1.46	16.85	20.38	1.76
1998	1.46	16.47	19.91	1.76
1999	1.45	15.97	19.09	1.73
2001	1.20	12.69	17.02	1.61
2002	1.37	14.32	16.47	1.58
2003	1.41	14.42	15.99	1.57
2004	1.45	14.75	15.93	1.57
2005	1.34	13.57	16.17	1.60
2006	1.38	14.04	15.84	1.56
2007	1.45	14.77	15.94	1.57
2008	1.48	15.13	16.08	1.57
2009	1.37	14.19	16.15	1.56
2011	1.03	11.20	16.04	1.48
2012	1.25	13.59	16.35	1.50
2013	1.22	13.37	16.40	1.50
2014	1.26	13.87	16.87	1.53
2015	1.05	11.42	16.55	1.52
2016	1.24	13.53	17.86	1.64
2017	1.58	16.88	17.23	1.61
2018	1.50	15.66	15.23	1.45
2019	1.47	14.97	14.65	1.44
2020	1.30	12.86	12.00	1.21
2021			10.62	1.07

The “TFR in Yearbook” are obtained by summing over the age-specific fertility rates provided in annual China Population Statistics Yearbook, which doesn’t provide the TFR, but provides age-specific fertility rates based on population sample surveys. The “Published births” are from annual Statistical communiqué of the People’s Republic of China National Economic and Social Development. With the “TFR in Yearbook” and base-year population, including age and sex structure, we obtained “Birth estimate” by population projection. Then, we calculated the “Estimated TFR” by Estimated TFR = TFR in Yearbook*Published births/Birth estimate

One noteworthy thing is that, before 2018, the projected TFRs were higher than the officially published figures. But for the three consecutive years 2018, 2019 and 2020, the projected TFRs were lower than the officially published. The reason for this reversal needs to be further explored.

## Conclusions and discussions

China’s fertility and births have attracted much attention with the implementation of its strict birth control policy, which has been highly controversial [55]. With the birth control policy is the fertility decline over the past decades, which has also been controversial in both its magnitude and its determinants [9, 56]. In this paper, we have presented the change in fertility in China using multiple measures.

Delayed marriage has a significant effect on fertility decline. This effect is reflected in the change in the age-specific fertility pattern, the increase in the mean age at childbearing, the declining TFR/TMFR ratio, and the standardization and decomposition of TFR. Delayed marriage contributes 0.42 during 1990–2000 and 0.17 during 2000–2010 to the decline in TFR. Age at first marriage is certain to increase, and delayed marriage and childbearing will further contribute to the fertility decline. China adopted the universal three-child policy in 2021. However, the people’s response suggests that the fertility rate will not increase significantly. China has now turned to comprehensive pronatalist policies nationwide, but whether these policies will help increase fertility remains to be seen.

Adjusted period fertility measures show that, after eliminating the tempo and parity structure effects, the adjusted TFR was below 1.5 in 2015, and the tempo- and parity-adjusted TFR for first births was 0.9 in 2015. Universal marriage and childbearing was the traditional norm for Chinese women, but this social pattern is changing. The proportion of childlessness is increasing, leading to a declining fertility rate for first births. Historical data in Europe show that the rising proportion of childless women played a significant role in further declining the already low fertility [57]. In East Asia, particularly Japan, the sharp rise in the proportion of childless women in the cohort born in the 1950s and 1960s resulted in CFR decline [58]. So the proportion of childlessness will have an important impact on the decline of future fertility in China.

Cohort fertility measures show a marked decline in CFR. During 1982–1990, the decline in CFR was mainly driven by the decline in fourth and higher-order births, during 1990–2000 mainly by the decline in second and third births, and during 2000–2015 mainly by the decline in second births. It is reasonable to predict that future fertility declines will be strongly driven by declines in first births or increases in the proportion of childlessness.

The indirect estimation techniques revealed TFR in the range of 1.5–1.6 for 2000–2010 and an average TFR of 1.5 for 2011–2020. Despite the slightly higher fertility rates in 2016 and 2017 due to the universal two-child policy introduced in 2016, China’s TFR fell to below 1.5 in 2018 and 1.3 in 2020. 2021 witnessed a further decline in birth numbers and the TFR was roughly estimated at 1.07. China’s TFR is unlikely to recover markedly in the near future, and it may remain at a very low level for a long time.

Scholarships reached a consensus that both the birth control policy and socioeconomic developments contributed to the fertility decline [9, 56, 59], that is, the fertility decline was both spontaneously brought about by the socioeconomic development and achieved by the enforcement of stringent birth control policies during the past decades. However, the magnitude in the fertility decline brought about by and the number of births averted by the birth control policy during the past decades has been quite controversial [9, 59–62] and remains a problem to be solved. With socioeconomic developments, the willingness to have more children has decreased markedly, and fertility intention and behavior have undergone fundamental changes [63]. The relaxation of stringent birth control by the introduction of universal two-child policy in 2016 and three-child policy in 2021 failed to raise the low fertility level. China has recently shifted to pronatalist policies to reverse the downward trend in fertility and births by eliminating past restrictive measures in births and by adopting a series of pronatalist measures, the effect of which remains to be seen.

The present study has certain limitations. Firstly, data quality is a concern in researching China’s fertility. We tried to adjust the TFR with an indirect estimation method and adjusted indicators. Nevertheless, the general description of this paper depends largely on the raw data from the census. Secondly, we computed many indicators but could not reach a generally accepted fertility level. The fertility level remains controversial. Thirdly, we did not analyze the data by residence or educational attainment, which may shed more light on the fertility trend in China with rapid urbanization and expansion of high education. Lastly, we did not include detailed data from the 2020 population census due to the data unavailability. Despite these limitations, the results presented here are still helpful in interpreting China’s fertility and population characteristics and may serve as a reference for future adjustment and improvement of China’s fertility policy.

## Data Availability

The respective statistical institutes provided the input data with the condition that only aggregated measures could be published. The derived dataset supporting the conclusions of the article is included within the article and its additional file.

## Appendix 1: The methods

### Period fertility measures

1. Period total fertility rate (TFR)

TFR is the average number of children expected to be born to a cohort of women as they pass through their childbearing years according to the age-specific fertility rates observed during the period. Designate ${}_{n}f_{a} \left( t \right)$ as the age-specific fertility rate in the year *t* for women aged (*a*, *a* + *n*) years, the $TFR$ in the year *t* can be expressed as:

1 $$TFR\left( t \right) = n\sum\limits_{a = \alpha ,n}^{\beta - n} {{}_{n}f_{a} \left( t \right)}$$

where *α* and *β* are the minimum and maximum childbearing ages, respectively.

2. Mean age at childbearing

Mean age at childbearing is a measure commonly used in fertility research. The mean age at childbearing in the year t $MAC\left( t \right)$ can be expressed as:

2 $$MAC\left( t \right) = \frac{{n\sum\nolimits_{a = \alpha ,n}^{\beta - n} {\left( {a + \frac{n}{2}} \right)_{n} f_{a} \left( t \right)} }}{{n\sum\nolimits_{a = \alpha ,n}^{\beta - n} {_{n} f_{a} \left( t \right)} }}$$

3. Total marital fertility rate (TMFR)

A common measure of marital fertility is the total marital fertility rate (TMFR). Designate ${}_{n}mf_{a} \left( t \right)$ as the age-specific marital fertility rate of the cohort of women aged $\left( {a,a + n} \right)$ years in year *t*, the total marital fertility rate *TMFR*(*t*) in year *t* can be expressed as:

3 $$TMFR\left( t \right) = n\sum\limits_{a = \alpha ,n}^{\beta - n} {{}_{n}mf_{a} \left( t \right)}$$

4. TFR/TMFR ratio

In China, the minimum legal marriage age for women is 20 years. In this study, the TMFR and TFR for women aged 20–49 years were compared. Designate ${}_{n}mp_{a} \left( t \right)$ as the proportion of married women to the total of women aged (*a*, *a* + *n*) years in year *t*, the TFR in year *t* can be expressed as:

4 $$TFR\left( t \right) = n\sum\limits_{a = \alpha ,n}^{\beta - n} {{}_{n}f_{a} \left( t \right)} = n\sum\limits_{a = \alpha ,n}^{\beta } {{}_{n}mf_{a} \left( t \right) \times {}_{n}mp_{a} \left( t \right)}$$

The TFR/TMFR ratio in year *t* can be expressed as:

5 $$\frac{TFR}{{TMFR}} = \frac{{n\sum\nolimits_{a = \alpha ,n}^{\beta - n} {_{n} mf_{a} \times_{n} mp_{a} } }}{{n\sum\nolimits_{a = \alpha ,n}^{\beta - n} {_{n} mf_{a} } }} = \sum\limits_{a = \alpha ,n}^{\beta - n} {\left( {\frac{{{}_{n}mf_{a} }}{{\sum\nolimits_{a = \alpha ,n}^{\beta - n} {_{n} mf_{a} } }}} \right) \times {}_{n}mp_{a} }$$

Equation (5) reveals that TFR/TMFR can be expressed as the average of age-specific proportions of married women weighted by the age-specific marital fertility rates. Thus, the TFR/TMFR ratio is a measure of the effect of the proportion of married women on TFR.

5. Standardization of TFR

Marriage postponement results in a decreased age-specific proportion of married women, thereby affecting the *TFR*. Taking ${}_{n}mp_{a} \left( t \right)$ in year *t* as the standard, the standardized *TFR* in year (*t* + *h*) can be expressed as follows:

6 $$TFR^{ + } \left( {t + h} \right) = n\sum\limits_{a = \alpha ,n}^{\beta - n} {{}_{n}mf_{a} \left( {t + h} \right) \times {}_{n}mp_{a} \left( t \right)}$$

The difference between the adjusted and unadjusted TFRs, $TFR^{ + } \left( {t + h} \right) - TFR\left( {t + h} \right)$, can be used to measure the effect of the variation in the age-specific proportion of married women on TFR.

6. Decomposition of TFR changes

The change in fertility during the period from year *t* to year *t* + *h* can be decomposed as:

7 $$\begin{aligned} & TFR\left( {t + h} \right) - TFR\left( t \right) \\ & \quad = n\sum\limits_{a = \alpha ,n}^{\beta - n} {\frac{{{}_{n}mp_{a} \left( {t + h} \right) \times {}_{n}mp_{a} \left( t \right)}}{2}} \times \left( {{}_{n}mf_{a} \left( {t + h} \right) - {}_{n}mf_{a} \left( t \right)} \right) \\ & \quad \quad + n\sum\limits_{a = \alpha ,n}^{\beta - n} {\frac{{{}_{n}mf_{a} \left( {t + h} \right) \times {}_{n}mf_{a} \left( t \right)}}{2}} \times \left( {{}_{n}mp_{a} \left( {t + h} \right) - {}_{n}mp_{a} \left( t \right)} \right) \\ \end{aligned}$$

The first and second terms on the right of Eq. (7) represent the effects of the changes in the marital fertility rate and the effect of the changes in the proportion of married women, respectively, on the TFR [1].

### Adjusted period fertility measures

1. Period Parity Progression Fertility

Based on existing studies [17, 18, 64], our study used census data to compute parity progression fertility measures as follows.

Designating $W_{a} \left( {t,i - 1} \right)$ as the number of women aged *a* years with a parity of i in *i *− 1 year *t*, $B_{a} \left( {t,i} \right)$ as the total number of children born to these women, the age-specific parity progression ratio can be computed using the following equations:

8 $$h_{a} \left( {t,i} \right) = \frac{{B_{a} \left( {t,i} \right)}}{{W_{a} \left( {t,i - 1} \right)}}$$

The period total parity progression fertility rate (PPPFR) of first and second births can be computed using the following equation:

9 $$\begin{aligned} PPPFR\left( {t,1} \right) = & \sum\limits_{a = 15}^{49} {\left[ {h_{a} \left( {t,1} \right)\prod\limits_{j = 15}^{a - 1} {\left( {1 - h_{j} \left( {t,1} \right)} \right)} } \right]} \\ PPPFR\left( {t,2} \right) = & \sum\limits_{a = 15}^{49} {\left[ {h_{a} \left( {t,2} \right)\prod\limits_{n = 15}^{a - 1} {h_{n} \left( {t,1} \right)\left( {\prod\limits_{j = 15}^{n - 1} {\left( {1 - h_{j} \left( {t,1} \right)} \right)\prod\limits_{k = n + 1}^{a - 1} {\left( {1 - h_{k} \left( {t,2} \right)} \right)} } } \right)} } \right]} \\ \end{aligned}$$

The period total parity progression fertility rate of third and higher-order births can be computed in the same manner.

2. Tempo- and parity-adjusted TFR

Bongaarts and Feeney (1998) [20] proposed an equation to account for the tempo effect:

10 $$TFR^{*} \left( t \right) = \sum\limits_{i} {TFR^{*} \left( {t,i} \right)} = \sum\limits_{i} {\sum\limits_{a} {\frac{{f_{a} \left( {t,i} \right)}}{{1 - r\left( {t,i} \right)}}} } = \sum\limits_{i} {\frac{{TFR\left( {t,i} \right)}}{{1 - r\left( {t,i} \right)}}}$$

where $TFR^{*} \left( {t,i} \right)$ is the tempo-adjusted TFR of *i*th birth in year *t*, and *r*(*t*, *i*) is the annual change rate in the mean age at childbearing and can be computed using the following equation:

11 $$r\left( {t,i} \right) = {{\left( {MAC\left( {t + 1,i} \right) - MAC\left( {t + 1,i} \right)} \right)} \mathord{\left/ {\vphantom {{\left( {MAC\left( {t + 1,i} \right) - MAC\left( {t + 1,i} \right)} \right)} 2}} \right. \kern-\nulldelimiterspace} 2}$$

Yamaguchi and Beppu (2004) [65] and Bongaarts and Feeney (2006) [66] proposed to express TFR using hazard rates as follows:

12 $$TFRp\left( t \right) = \sum\limits_{i} {TFRp\left( {t,i} \right)} = \sum\limits_{i} {\left\{ {1 - \exp \left[ { - \sum\limits_{a} {p_{a} \left( {t,i} \right)} } \right]} \right\}}$$

where $p_{a} \left( {t,i} \right)$ is the hazard rate of the *i*th birth of women aged *a* years in year *t* and is computed by dividing the number of women aged *a* years giving the *i*th birth in year *t* by the number of women aged *a* years with parities less than *i* in year *t*.

Bongaarts and Feeney (2006) [66] proposed tempo- and parity-adjusted total fertility, $TFRp^{*} \left( t \right)$ which is expressed as:

13 $$TFRp^{*} \left( t \right) = \sum\limits_{i} {TFRp^{*} \left( {t,i} \right)} = \sum\limits_{i} {\left\{ {1 - \exp \left[ { - \sum\limits_{a} {\frac{{p_{a} \left( {t,i} \right)}}{{1 - r\left( {t,i} \right)}}} } \right]} \right\}} .$$

### Cohort fertility measures

1. The proportion of women aged 45–49 with at least N live births

Designate $W_{i}^{c}$ as the number of women in a given cohort *c* with at least *i* live births, and $W^{c}$ as the number of women in cohort *c*, the proportion of women $R_{i}$ with at least *i* live births is expressed as:

$$R_{i} = \frac{{W_{i}^{c} }}{{W^{c} }}$$

2. Completed cohort fertility rate

Completed cohort fertility rate (*CFR*) is the arithmetic mean of the number of children born to a cohort of women who have completed childbearing. The *CFR* is expressed as:

14 $$CFR = \sum\limits_{i} {CFR_{i} } = \sum\limits_{i} {R_{i} }$$

where $CFR_{i}$ is the *CFR* for the *i*th birth.

3. Cohort parity progression ratio

Designate the parity progression ratio of a given cohort from the (*i*-1)th birth to the *i*th birth as $PPR_{i - 1,i}$, the parity progression ratios to the first birth, from the (*i *− 1) birth to the *i*th birth, and from the *n*th birth to the (*n* + 1)th and higher-order births can be expressed as:

15 $$\begin{aligned} & PPR_{0,1} = CFR_{1} \\ & PPR_{i - 1,i} = \frac{{CFR_{i} }}{{CFR_{i - 1} }},\;i > 1 \\ & PPR_{n + ,(n + 1) + } = \frac{{CFR_{(n + 1) + } }}{{CFR_{n + } }} \\ \end{aligned}$$

where $CFR_{n + }$ and $CFR_{(n + 1) + }$ are the CFR for the *n*th and higher-order births and the (*n* + 1)th and higher-order births, respectively.

The CFR and parity progression ratio of a given cohort satisfies the following relationship [19]:

16 $$CFR = \sum\limits_{i} {CFR_{i} } = \sum\limits_{i} {\prod\limits_{j = 1}^{i} {PPR_{j - 1,j} } }$$

4. Decomposition of cohort CFR variations

Zeman et al. (2018) [25] expressed the relationship between cohort CFR and parity progression ratio as follows:

17 $$\begin{aligned} CFR = & PPR_{0,1} + PPR_{0,1} \times PPR_{1,2} + PPR_{0,1} \times PPR_{1,2} \times PPR_{2,3} \\ & \quad + PPR_{0,1} \times PPR_{1,2} \times PPR_{2,3} \times PPR_{3,4} \\ & \quad + PPR_{0,1} \times PPR_{1,2} \times PPR_{2,3} \times PPR_{3,4} \times \frac{{PPR_{4 + ,5 + } }}{{1 - PPR_{4 + ,5 + } }} \\ \end{aligned}$$

The difference in the CFRs of cohorts *c*1 and *c*2 can then be decomposed as [25]:

18 $$CFR^{c2} - CFR^{c1} = dPPR_{0,1}^{c1,c2} + dPPR_{1,2}^{c1,c2} + dPPR_{2,3}^{c1,c2} + dPPR_{3,4 + }^{c1,c2}$$

The four terms on the right of Eq. (18) represent the effects of the changes in the progression ratios from the 0 parity to first birth, from first to second births, from second to third births, and from third to fourth and higher-order births, respectively, on the change in CFR. This method can determine which order of birth has the biggest contribution to cohort CFR decline [25].

### Indirect estimation of fertility

We included the following indirect methods: variable-r method, P/F ratio methods, reverse survival method, and projection simulation method.

1. Variable-r method

The following is an illustration of estimating fertility using the variable-r method:

The net reproduction rate of a population, *NRR*, can be expressed as:

19 $$NRR = \int_{\alpha }^{\beta } {v\left( x \right) \times e^{{\int_{0}^{x} {r\left( a \right){\text{d}}a} }} {\text{d}}x}$$

where *v*(*x*) is the ratio of the girl babies born to women aged *x* years to the total newborn girls, and *r*(*a*) is the growth rate of the population of women aged *a* years between two time points, *t*_1_ and *t*_2_. Using this method, TFR can be expressed as:

20 $$TFR = \frac{{\left( {1 + SRB} \right) \times NRR}}{{p\left( {\overline{m}} \right)}}$$

where $p\left( {\overline{m}} \right)$ is the probability for a newborn girl living to the mean age at childbearing, and SRB is the sex ratio at birth.

2. P/F ratio method

The P/F ratio method with one single census data and with two census data was introduced by the United Nation (1983) [54]. For the adjustment coefficient of P/F ratio, we choose the average value of P/F ratio at 25–29 and P/F ratio at 30–34 when applying this method to a single census and two censuses.

3. Reverse survival method

In this study, the newborn population in 2000 was estimated reversely using 2010 census data, the newborn population in 1990 was estimated using the 2000 census data using the reverse survival method, and the newborn population in 1982 was estimated reversely using the 1990 census data, by using the following equations:

$$B_{2000} = \frac{{P_{10}^{2010} }}{{L_{10} /l_{0} }}$$

21 $$B_{1990} = \frac{2}{3} \times P_{10}^{2000} \times \left( {\frac{{l_{0} }}{{\frac{2}{3}L_{10} + \frac{1}{3}L_{11} }}} \right) + \frac{1}{3} \times P_{11}^{2000} \times \left( {\frac{{l_{0} }}{{\frac{2}{3}L_{10} + \frac{1}{3}L_{11} }}} \right)$$

$$B_{1982} = \frac{{P_{8}^{1990} }}{{L_{8} /l_{0} }}$$

where $l_{0}$ is the initial population in the life table, *L*_*a*_ is the number of person-years of the population aged *a* years in the life table living from *a* years to (*a* + 1) years, and $P_{a}^{y}$ is the population aged *a* years in year *t*. The ratio of the newborn population obtained through the reverse survival analysis to that registered by census was then used to adjust the fertility rate.

4. Projection simulation method

Both the census data and annual population sample survey data of the National Bureau of Statistics include age-specific fertility rates. The survey TFR derived by summarizing the age-specific fertility data is rather low. With a given year as the baseline, annual newborn populations can be simulated through population projection using survey fertility rates. Then the ratio of published annual birth numbers by NBS to the derived annual newborn population was computed. These ratios were then used as coefficients to adjust the survey fertility rates published by the NBS.

## Appendix 2

See Tables 9 and 10.

**Table 9:** TFRs by year

Year	TFR	TFR_1_	TFR_2_	TFR_3+_
1982	2.61	1.18	0.64	0.79
1990	2.25	1.01	0.72	0.52
1995	1.43	0.96	0.36	0.11
2000	1.22	0.87	0.29	0.07
2005	1.34	0.89	0.38	0.06
2010	1.19	0.73	0.38	0.08
2015	1.05	0.56	0.42	0.08

Data source: The results for 1982–2015 were computed using tabulated age-specific fertility data from the censuses and 1% population sample surveys during the same period

**Table 10:** Period parity progression fertility rate

Year	PPPFR_1_	PPPFR_2_	PPPFR_3_	PPPFR_4_	PPPFR_5+_	PTPPFR
1982	1.00	0.88	0.44	0.14	0.08	2.54
1990	0.99	0.71	0.29	0.09	0.04	2.11
1995	0.96	0.43	0.14	0.17		1.70
2000	0.97	0.33	0.03	0.00	0.00	1.33
2005	0.98	0.41	0.04			1.42
2010	0.98	0.37	0.05			1.39
2015	0.86	0.34	0.08			1.28

Data source: The 1982, 1990, and 2000 data come from Wang (2004) [48]; the 1995 data come from Guo (2000) [22]; the 2005 and 2010 data come from Guo (2013) [49]; the 2015 results were computed using the 1% population sample survey in 2015
